# Aminopeptidase N: the glucocorticoid gateway linking chronic stress to ferroptosis resistance in liver cancer

**DOI:** 10.1172/JCI202410

**Published:** 2026-02-16

**Authors:** Maowu Luo, Weibo Luo

**Affiliations:** 1 Department of Pathology and; 2 Department of Pharmacology, UT Southwestern Medical Center, Dallas, Texas, USA.

## Abstract

Chronic stress triggers a range of physiological responses that could dysregulate the immune system and metabolic processes, thereby increasing susceptibility to various diseases. In this issue of the *JCI*, Wu et al. identified a metabolic bridge between chronic stress and liver cancer progression. Chronic stress–induced glucocorticoids promoted aminopeptidase N (ANPEP) expression and subsequent reprogramming of amino acid metabolism, leading to increased liver cancer growth and metastasis. ANPEP facilitated stabilization of the cystine-glutamate transporter system X_c_^–^ and increased l-cystine influx, thereby enhancing cellular antioxidant capacity to prevent ferroptosis. Silencing ANPEP in combination with sorafenib treatment showed a synergistic inhibitory effect on liver cancer progression. These findings uncover ANPEP as a valuable target for therapeutic interventions to treat patients with liver cancer experiencing chronic stress.

## Chronic stress and liver cancer

**Figure 1:**
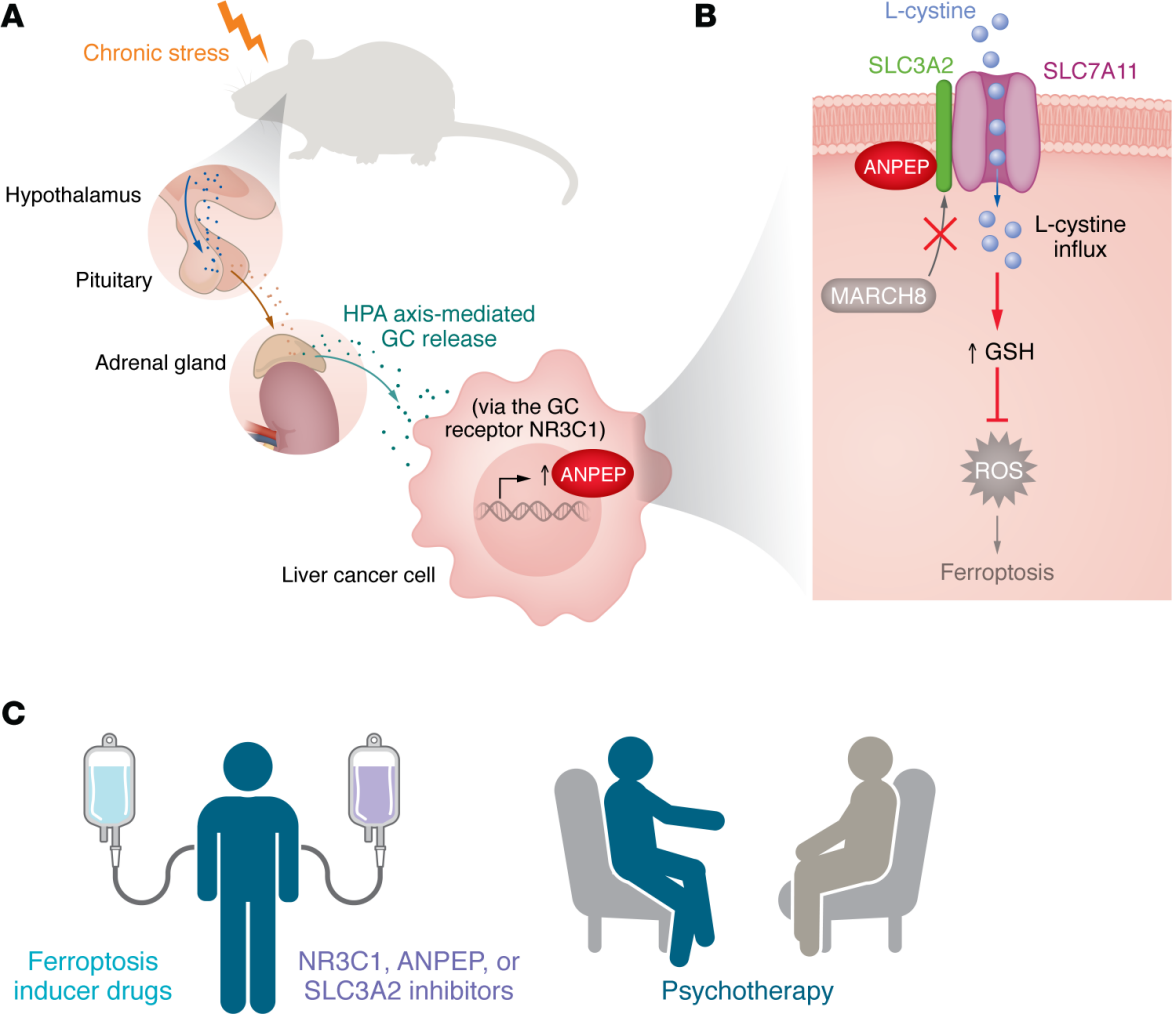
Chronic stress–induced ANPEP drives ferroptosis resistance in liver cancer. (**A**) Wu et al. (11) modeled chronic stress–mediated activation of the hypothalamic-pituitary-adrenal axis using chronic restraint stress. Stress-induced GCs mediated nuclear translocation of NR3C1, activating *ANPEP* transcription in liver cancer cells. (**B**) Wu et al. showed that ANPEP interacted with SLC3A2, a transmembrane subunit of the l-cystine/glutamate antiporter system X_c_^–^ (which also contains the subunit SLC7A11 shown in the figure). This interaction prevented SLC3A2 from undergoing MARCH8-mediated lysosome-dependent degradation, thereby increasing synthesis of the intracellular antioxidant GSH and protecting liver cancer cells against ferroptosis. (**C**) Potential therapeutic strategies for liver cancer may include targeting the NR3C1/ANPEP/SLC3A2 axis or implementing psychotherapy to reduce stress, sensitizing tumors to ferroptosis-inducing anticancer therapies.

Liver cancer develops after chronic liver injuries, including cirrhosis, hepatitis B or C virus infection, alcohol use, and metabolic dysfunction–associated steatohepatitis (1). It is a malignant disease with a steadily increasing incidence and mortality in the United States (2). Recent clinical studies reveal that chronic stress is one of the factors associated with poor survival outcomes in patients with liver cancer (3). Chronic stress, a common psychological condition in modern society, arises from prolonged exposure to stressors in daily life and has detrimental effects on physical and mental health (4). In contrast with acute stress, chronic stress sustains a prolonged activation of the hypothalamic-pituitary-adrenal axis and the sympathetic nervous system, resulting in a continuous release of glucocorticoids (GCs) and catecholamines (Figure 1A) (5). Previous studies have shown that these stress hormones promote cancer development and metastasis by regulating angiogenesis, metabolism, DNA damage, inflammation, and immunosuppression in various preclinical cancer models (6–10). However, the contribution of chronic stress to liver cancer and the mechanisms involved remain poorly defined.

In this issue of the *JCI*, Wu et al. (11) identified a detailed mechanism that elucidated how stress hormones promoted liver cancer progression and metastasis (Figure 1, A and B). The authors used a chronic restraint stress murine model that dramatically elevates serum corticosterone (the murine GC), demonstrating a remarkable effect of chronic stress on liver cancer progression in this animal model. Utilizing multiomics, including metabolomics and RNA sequencing, they identified a consequential rewiring of amino acid metabolic pathways, with *Anpep* emerging as the most significantly upregulated gene. *Anpep* encodes aminopeptidase N, an enzyme involved in amino acid metabolism. Next, in two human liver cancer cell lines, Wu et al. uncovered the key transcriptional mechanism underlying ANPEP upregulation: Upon GC stimulation, nuclear receptor subfamily 3 group C member 1 (NR3C1) directly bound to the *ANPEP* promoter and enhanced its transcription (Figure 1A). The GC/NR3C1/ANPEP axis acts as a molecular bridge, translating systemic chronic stress into a specific, actionable metabolic command within the liver cancer cell. This finding transforms ANPEP from a mere prognostic marker into a GC-responsive metabolic gateway in liver cancer (12, 13). The observation that ANPEP was also upregulated by chronic stress in colon cancer suggests that this mechanism may represent a generalizable pathway linking chronic stress to tumor-specific metabolic reprogramming across multiple cancer types (11).

## ANPEP upregulation promotes ferroptosis resistance in liver cancer

Cancer cells inherently face high levels of oxidative stress because of their rapid proliferation, high metabolic activity, and harsh tumor microenvironment (14). Oxidative stress causes lipid peroxidation and triggers ferroptosis, an iron-dependent nonapoptotic form of cell death (15). While the liver serves as a major iron reservoir (16), liver cancer cells adapt to redox imbalance by activating antioxidant defense systems, thereby limiting ferroptosis (17). Glutathione (GSH), an abundant cellular antioxidant, plays a pivotal role in this process (18). The authors convincingly showed that ANPEP, once transcriptionally activated by chronic stress, promoted GSH synthesis to reduce reactive oxygen species and lipid peroxidation in liver cancer cells, thereby preventing ferroptosis and conferring a critical survival advantage (11).

GSH is a tripeptide composed of three amino acids: l-cysteine, l-glutamate, and l-glycine. The availability of l-cysteine is the rate-limiting step in GSH synthesis. The stable influx of l-cystine via the cystine/glutamate antiporter system X_c_^–^ and its subsequent oxidation to l-cysteine are critical to sustain high GSH levels (18, 19). In the present work, Wu et al. showed that ANPEP interacted with and stabilized solute carrier family 3 member 2 (SLC3A2) protein (11), a transmembrane subunit of system X_c_^–^ (Figure 1B). This interaction prevented the E3 ubiquitin ligase membrane associated ring-CH-type finger 8 (MARCH8) from binding and ubiquitinating SLC3A2, thereby inhibiting its lysosomal trafficking and degradation (Figure 1B). Through its interaction with SLC3A2, ANPEP is also associated with SLC7A11, another subunit of system X_c_^–^ responsible for l-cystine influx (19). Consequently, ANPEP promoted chronic stress–induced l-cystine uptake, leading to increased intracellular l-cysteine and elevated GSH levels in liver cancer cells (Figure 1B). This signaling cascade protected liver cancer cells against oxidative stress and ferroptosis (Figure 1B).

Experimental therapeutic studies showed that genetic inhibition of *ANPEP* or *SLC3A2* prevented chronic stress–induced liver cancer xenograft growth and metastasis in mice (11). Notably, ANPEP overexpression reduced liver cancer sensitivity to sorafenib, an FDA-approved liver cancer drug known to induce ferroptosis (20), whereas ANPEP silencing enhanced sorafenib’s antitumor efficacy in mice by increasing intratumoral lipid peroxidation and ferroptosis (11). These findings provide a strong rationale for targeting cellular metabolism in combination with standard therapy to treat liver cancer (Figure 1C).

## Impact and future directions

The findings by Wu et al. provide innovative insights into psychological regulation of metabolism–cell fate crosstalk that drives cancer development. The chronic stress signature the authors developed to predict survival outcomes in patients with liver cancer represents a clinically important prognostic tool. More importantly, this study highlights the potential of specific ANPEP or SLC3A2 inhibitors as therapeutic interventions for liver cancer, particularly for patients with elevated stress hormone levels.

Although comprehensive in its identification of a pathway linking chronic stress to liver cancer progression, the study raises several outstanding questions to be addressed in the future. (a) People are exposed to various forms of psychological stress in modern society, and chronic restraint stress may model specific types of stress. It remains unclear whether ANPEP-mediated signaling similarly contributes to tumor progression and metastasis under other forms of psychological stress, such as those modeled by social isolation. (b) ANPEP is a transmembrane peptidase that plays diverse roles in peptide digestion, antigen presentation, and cell signaling (21). Future investigation should determine whether its enzymatic activity, which hydrolyzes amino acids and peptides, is responsible for the metabolic reprogramming observed in this study. (c) Amino acids serve as essential fuels for tumor progression (22). Considering that multiple amino acid pathways are altered by chronic stress and ANPEP (11), a comprehensive understanding of the ANPEP-dependent metabolic landscape in response to chronic stress may provide critical insights into how chronic stress reshapes tumor metabolism and identify additional therapeutic opportunities. (d) Psychotherapy has been shown to alleviate chronic stress (23). It would be interesting to study whether psychotherapy can improve outcomes in patients with liver cancer receiving standard therapies (Figure 1C).

In conclusion, in this *JCI* paper, Wu et al. elucidate a fundamental metabolic connection between chronic stress and liver cancer progression. By identifying the ANPEP/SLC3A2 axis as a key mediator of chronic stress–induced ferroptosis resistance and liver cancer progression, the study offers a major conceptual advance as well as a strong rationale for developing new therapeutic strategies for liver cancer.

## Funding support

- Grants from Department of Defense (HT9425-23-1-0863, HT9425-24-1-0538, and HT9425-24-1-0702).
- CPRIT (grant RP220178).
- CPRIT Scholar in Cancer Research (WL).
- Alex’s Lemonade Stand Foundation (grant 1433529).

## Version 1 02/16/2026

Electronic publication
